# Paving the way for universal medical student training in serious illness communication: the Massachusetts Medical Schools’ Collaborative

**DOI:** 10.1186/s12909-022-03702-2

**Published:** 2022-09-01

**Authors:** Jennifer A. Reidy, Melissa A. Clark, Harris A. Berman, Stephanie H. Chan, Atul A. Gawande, Jocelyn Streid, Tamara Vesel, Megan E. Young, April Zehm, Kristen G. Schaefer

**Affiliations:** 1 UMass Memorial Medical Center, 55 Lake Avenue North, Worcester, MA 01655 USA; 2 UMass Chan Medical School, 55 Lake Avenue North, Worcester, MA 01655 USA; 3 Brown University School of Public Health, 121 S Main St, Providence, RI 02903 USA; 4 Tufts University School of Medicine, 136 Harrison Ave, Boston, MA 02111 USA; 5 Massachusetts Coalition for Serious Illness Care, 101 Huntington Ave, Ste 1300, Boston, MA 02199 USA; 6 Blue Cross Blue Shield of Massachusetts, 101 Huntington Ave, Ste 1300, Boston, MA 02199 USA; 7 Brigham and Women’s Hospital, 75 Francis St, Boston, MA 02115 USA; 8 Harvard Medical School, 25 Shattuck St, Boston, MA 02115 USA; 9 Harvard T.H. Chan School of Public Health, 677 Huntington Ave, Boston, MA 02115 USA; 10 Ariadne Labs, 401 Park Dr, 3rd Floor, Boston, MA 02215 USA; 11 Tufts Medical Center, 800 Washington St, Boston, MA 02111 USA; 12 Boston University School of Medicine, 72 E Concord St, Boston, MA 02118 USA; 13 Medical College of Wisconsin, 8701 W Watertown Plank Rd, Wauwatosa, WI 53226 USA; 14 Froedtert Memorial Lutheran Hospital, 9200 W. Wisconsin Ave, Milwaukee, WI 53226 USA; 15 Care Dimensions, 75 Sylvan Street, Suite B-102, Danvers, MA 01923 USA

**Keywords:** Serious illness communication, Palliative care, Undergraduate medical education, Competencies

## Abstract

**Background:**

Patients with serious illness look to their clinicians for discussion and guidance on high-stakes treatment decisions, which are complex, emotional and value-laden. However, required training in serious illness communication is rare in U.S. medical schools, with efforts at curricular reform stymied by competing institutional demands, lack of resources and accreditation requirements. We describe an approach to building and scaling medical student training in serious illness communication through the creation of a statewide collaborative of medical schools.

**Methods:**

The Massachusetts Medical Schools’ Collaborative is a first-of-its-kind group that promotes longitudinal, developmentally-based curricula in serious illness communication for all students. Convened externally by the Massachusetts Coalition for Serious Illness Care, the collaborative includes faculty, staff, and students from four medical schools.

**Results:**

The collaborative started with listening to member’s perspectives and collectively developed core competencies in serious illness communication for implementation at each school. We share early lessons on the opportunities, challenges and sustainability of our statewide collective action to influence curricular reform, which can be replicated in other topic areas.

**Conclusions:**

Our next steps include curriculum mapping, student focus groups and faculty development to guide successful and enduring implementation of the competencies to impact undergraduate medical education in Massachusetts and beyond.

## Background

People with serious illness face many decisions, both personal and medical, that can be frightening, difficult, and confusing, and they rely on their care teams to clearly communicate prognosis and treatment options over the course of their illness. Studies have shown that patients who have conversations with their clinicians about prognosis, medical and personal goals, and treatment preferences are more likely to receive goal-concordant care and experience better quality of life [1]. However, The John A. Hartford Foundation’s 2016 survey of doctors found 46 percent of physicians report not knowing what to say during conversations with seriously ill patients, and only 29 percent felt they received the necessary communication skills training to break difficult news, explore goals of care and navigate end-of-life decisions [2].

In U.S. medical schools, training in serious illness communication (along with other essential palliative care concepts and skills) is lacking, sporadic and mostly elective [3]. Aside from a few outstanding examples of longitudinal, integrated palliative care curricula (such as the University of Rochester [4] and Yale Medical School [5]), most U.S. medical students learn serious illness communication “on the fly” by observing clinicians with varying degrees of skill, and without receiving specific guidance or feedback. While the Liaison Committee on Medical Education directs medical schools to teach aspects of end-of-life care, the Association of American Medical Colleges explicitly recommends training in palliative care for all stages of illness, prognostic reasoning, shared decision-making and communication of difficult news within clinical skills curricula [6]. Medical schools face competing demands and most lack strategies and resources to integrate serious illness communication training into their required longitudinal curriculum, despite national expert-consensus competencies published in 2014 that “raised the bar” for palliative care education standards in undergraduate medical education [7].

The slow pace of curricular reform at medical schools may be related to their traditional organizational hierarchies, often constrained by resources and accreditation guidelines. In the business literature, organizational change expert John Kotter describes the strategy of a “dual operating system” or a network of energized volunteers who work within and alongside traditional hierarchies to execute a shared vision of institutional change [8]. This collaborative approach can accelerate organization change via an engine of peer support and collective expertise to drive innovation with shared results.

In this report, we describe the creation of a statewide collaboration of faculty, administrators, and students within and alongside four medical schools to promote serious illness communication skills as essential and required for all future physicians.

Of note, this approach is applicable to any area of curricular reform. In 2015, our schools convened to confront the prescription opioid epidemic at the request of the state governor and developed shared educational competencies on safer opioid prescribing and diagnosis and treatment of substance use disorder to guide undergraduate instruction [9]. In this report, we go beyond a strategy of educational competencies alone and describe our efforts to build a sustainable collaborative to address a different gap – serious illness communication training – for all students. We share our first steps in defining our mission, scope of work and educational competencies, which set the stage for curriculum mapping, novel educational design, and faculty development as next steps.

## Methods

### About the Massachusetts medical schools’ collaborative

The Massachusetts Coalition for Serious Illness Care (the Coalition) is a group of over 125 member organizations in Massachusetts dedicated to ensuring that everyone gets care that supports what matters most to them, especially if they are seriously ill. In response to the important gaps and barriers in medical education, Harris Berman, MD, former dean of the Tufts University School of Medicine, and Atul Gawande, MD, founder and co-chair of the Coalition, brought together the deans of Massachusetts’ four medical schools to commit to a goal of training students in serious illness communication as a graduation requirement.

**Fig. 1:**
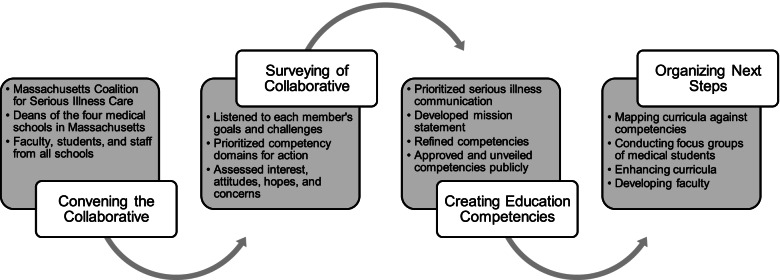
Roadmap of the Massachusetts Medical Schools’ Collaborative in Serious Illness Communication

This new group on serious illness communication—the Massachusetts Medical Schools’ Collaborative—includes expert faculty in palliative care and geriatrics, administrative leaders, and students from Boston University School of Medicine, Harvard Medical School, Tufts University School of Medicine and UMass Chan Medical School. The Coalition was and remains the collaborative’s external sponsor and convener. The collaborative started with each school performing an informal scoping review of its current palliative care curricula to share with the group. One of the authors (MC), conducted an anonymous survey of collaborative members, including faculty and associate deans (*n* = 16), to gauge participants’ interest, attitudes, hopes and concerns about the collaborative concept. The members met for a series of in-person meetings at Ariadne Labs in Boston to listen to each other’s perspectives, goals, and challenges in advancing their palliative care curricula and committed to helping accelerate each school’s efforts via peer mentorship and collective action. Two of the authors (AAG, JAR) facilitated three full-group discussions over nine months to define the collaborative’s mission and scope of work, starting with development of educational competencies. In anticipation of implementing these competencies, the group created a roadmap including creation of working groups on formal curriculum mapping, student engagement and faculty development (Fig. 1).

## Results

### Outcomes of the Massachusetts medical schools’ collaborative

The collaborative decided to adapt previously published medical student palliative care competencies as a foundation for the project [7]. Developed by consensus among national palliative care experts, these competencies had previously been adapted from the field’s fellowship competencies to meet the educational needs of residents and medical students. Our anonymous survey of collaborative members, including faculty and associate deans (*n* = 16), resulted in a clear priority for serious illness communication (Table 1). The survey also assessed participants’ views on the collaborative concept and goals. The majority (81%) preferred a phased project starting with communication and later focusing on other domains such as pain and symptom management and psychosocial, spiritual and cultural aspects of care. The same majority (81%) reported a preference for standardizing competencies across institutions while smaller percentages thought that curriculum design (38%), training faculty in the curriculum (31%) and evaluation (56%) should be standardized across institutions. Participants perceived a number of potential barriers to the collaborative, including differences in resources and cultures among the schools, and the ability to achieve consensus on the group’s priorities and outcomes. Participants named the collaborative’s strengths as strong expertise from nationally recognized faculty leaders and the Coalition, and a potential national model for accelerating palliative care skills in medical education as an innovative statewide effort.

**Table 1:** Competence and Competency Domains for Undergraduate Medical Students (*n* = 16)

	Median Ranking	Average Ranking	Standard Deviation
**Domains related to palliative care competencies (1 = most important, 5 = least important)**			
Communication	1.0	1.5	1.0
Pain and symptom management	3.0	2.9	1.1
Palliative care principles and practice	3.0	2.9	1.2
Psychosocial, spiritual, and cultural aspects of care	3.5	3.6	1.0
Terminal care and bereavement	5.0	4.1	1.4

The collaborative next appointed a working group with representatives from each school to adapt and refine competencies in serious illness communication. The working group, which communicated via virtual meetings and email discussions, crafted a mission statement: “*upon graduation, medical students will have acquired the foundational knowledge, skills and inspiration to engage as residents in goal-oriented conversations with seriously ill patients, with commitment to lifelong learning and deliberate practice*.” After multiple revisions, the working group presented a set of five competencies (Table 2), which were unanimously approved by the collaborative for implementation at each school. The collaborative unveiled the competencies to the public at the Coalition’s annual summit in 2018 alongside student video testimonials [10] which attracted significant media attention [11–14].

### Description of competencies

The competencies (Table 2) contain the building blocks for training future physicians in complex, emotional, high-stakes conversations with seriously ill patients to better align medical care with their goals. The first competency emphasizes that students must start by assessing patients’ and families’ understanding of illness and eliciting their priorities and values as critical context for developing the medical plan of care. The second competency defines the skill of responding to strong emotion inherent in these conversations as a critical tool for cultivating connection and means to offer comfort and support to people throughout serious illness. The third competency highlights cognitive frameworks as students observe and then practice conversations about prognosis, goals and treatment options. Of note, collaborative members discussed and agreed that students should first practice these conversations in low stakes learning environments such as role play and simulation, followed by supervised, incremental skills acquisition in real-world encounters (such as eliciting patients’ perspectives or asking about their hopes and fears) as they prepare for residency. The fourth competency aims to equip students early in their careers with strategies for self-awareness and reflective practice essential for effective communication, and the resiliency skills to reduce burnout in future physicians. Finally, the fifth competency requires students to know and explain the principles and practice of palliative care and hospice—including how they are distinct from each other—as essential to formulating goal-concordant plans with patients, families, and clinical colleagues.

**Table 2:** Medical Student Competencies in Palliative Care Domain #1: Serious Illness Communication

Mission statement: Upon graduation, medical students will have acquired the foundational knowledge, skills and inspiration ton engage as residents in goal-ariented conversations with seriously ill patients, with commitment to lifelong learning and deliberate practice
Competency #1: Explores patient and family understanding of illness, concerns, values, and goals in order to develop goal-concordant treatment palns across settings of care
Competency #2: Demonstrates effective approaches to exploring and responding to strong emotions in patients and families facing serious illness
Competency #3: Applies a patient-centered framework to sharing difficult news, exploring pain and symptom burden, assessing prognostic awareness, discussing resuscitation preferences, and describing care at end of life
Competency #4: Demonstrates awareness of one’s own emotions and attitudes, and coping strategies for managing stress and uncertainty when caring for seriously ill patients
Competency #5: Defines and explains the philosophy and role of palliative care and differentiates hospice from palliative care

## Discussion

As Kotter describes in his concept of a “dual operating system,” the Massachusetts Medical Schools’ Collaborative was designed as an informal, energized network of faculty, administrators and students with a shared mission to advance serious illness communication training in medical education, with ongoing support from an influential external sponsor to help with project management and visibility. This first-of-its-kind collaborative aims to elevate and promote curricular development in serious illness communication at each school by leveraging peer expertise and shared work, starting with the standardized educational competencies. Our model’s structure and process are broadly applicable to any topic of curricular reform relevant to all medical schools.

Key elements in creating and sustaining the collaborative include having the Coalition as an external sponsor and convener driving the work; administrative champions at each school with influence on curricular priorities and decisions; faculty leaders with content expertise and broad relationships with other faculty within their schools; and student leaders with passion to promote the collaborative’s mission among their peers via word of mouth, social media and formation of student interest groups, which cultivate future student leaders. The collaborative’s success requires equal, respectful partnerships grounded in trust as well as efficient work processes among the multi-school membership.

Amidst our early success, the biggest challenges to the collaborative’s sustainability have included lack of protected time and funding for faculty leaders; competing priorities at each school, including accreditation visits and institutional shifts in curricular delivery, and continuity of student participation over time. Counteracting benefits of the informal network have been flexibility and lack of hierarchy where members share leadership and commit to focused areas of work. The creation and promotion of the collaborative itself has attracted a broader swath of administrators and faculty, eager to understand the work of their peers and excel with innovative curricula that fulfill accreditation requirements. Finally, this model has empowered student leaders with better access to curricular reform efforts and sponsored “seats at the table” within formal hierarchies of medical education. Examples of strategies for student engagement have included formation of student interest groups in palliative care and geriatrics; general recruitment (especially to first- and second- year students with fewer clinical demands) via emails and social media, and individual mentoring of students who incorporate this work into required or elective scholarly projects over their medical school careers.

## Conclusions and next steps

This report describes our collaborative’s formation, mission, and scope of work, including our first steps in creating shared educational competencies. We have since developed and implemented a standardized curriculum mapping process to these competencies. The mapping process will provide each school with data on educational gaps and opportunities to strategically tailor its curricula to students’ professional development, including the need for creation of milestones and assessment tools. With repeated use, the mapping process can detect curricular change over time.

With funding from the Coalition, the collaborative also hired an outside moderator to conduct focus groups of fourth-year students to understand their experiences and attitudes about serious illness communication. Finally, the deans of each medical school and the Coalition have committed to investing in shared faculty development, with the future goal of developing a community of educators with a common pedagogy and peer mentorship.

To date, we have working groups of faculty, students and administrators who are analyzing data from the curriculum mapping process and student focus groups and plan to disseminate our results in peer-reviewed publications. Meanwhile, the collaborative and its members disseminate the competencies and roadmap via strategic messaging aimed at local, state, and national audiences, including website development, electronic newsletters, grand rounds and virtual presentatons [15].

With a national shortage of palliative care clinicians and geriatricians to care for an aging population, it is mandatory to find innovative solutions to train all physicians in effective, high-quality communication with seriously ill patients. We urge other medical schools to incorporate these competencies, form partnerships based on local resources, and empower faculty and student leaders to “raise the bar” for all graduating medical students.

## Data Availability

The datasets used and/or analyzed during the current study are available from the corresponding author on reasonable request.
